# Structural insights into sGC activation by different activators

**DOI:** 10.1186/1471-2210-11-S1-O11

**Published:** 2011-08-01

**Authors:** Focco van den Akker, Faye Martin, Vijay Kumar, Xiaolei Ma, Johannes-Peter Stasch, Martina Schaefer, Padmamalini Baskaran, Annie Beuve, Pete W van Dunten

**Affiliations:** 1 Department of Biochemistry/RT500, Case Western Reserve University, Cleveland, OH 44106, USA; 2 Cardiovascular Research, Bayer Schering Pharma AG, Wuppertal D-42096, Germany; 3 Department of Pharmacology and Physiology, New Jersey Medical School, U. Of Medicine and Dentistry of New Jersey, Newark, NJ 07103, USA; 4 Stanford Synchrotron Radiation Laboratory, Stanford University, Menlo Park, CA 94025, USA

## Background

The soluble guanylyl cyclase (sGC) is a key enzyme involved in the production of the second messenger cGMP. Due its cardiovascular relevance, sGC has been the target of numerous drug discovery efforts leading to the development of many different activators and stimulators with pharmaceutical potential. One of such compounds is the sGC activator Cinaciguat (BAY 58-2667) which is in clinical trials for acute decompensated heart failure.

## Results

We present here our latest structure-function studies related to sGC activation by different sGC activators. The activator complex structures reveal an intriguing mode of heme mimicry by these compounds. The observed activation conformational changes center around the αF helix that contains the H105 residue normally held in place by the heme moiety in the absence of the activators.

## Conclusion

The structural insights gained from the complex structures could be used for further optimization and development of sGC activators.

